# Impact of COVID-19 on the Research Career Advancement of Health Equity Scholars from Diverse Backgrounds

**DOI:** 10.3390/ijerph20064750

**Published:** 2023-03-08

**Authors:** Adriana Báez, Muhammed Y. Idris, Kimberly Lawson, Mohamed Mubasher, Yulia Strekalova, Keith Green, Priscilla Pemu, Jonathan K. Stiles, Martiza Salazar, Alexander Quarshie, Lee S. Caplan, Ernest Alema-Mensah, Thomas Pearson, Jessica Faupel-Badger, Jeffrey A. Engler, Elizabeth O. Ofili

**Affiliations:** 1 Departments Pharmacology and Otolaryngology, School of Medicine, University of Puerto Rico, San Juan, PR 00936, USA; 2 Departments of Medicine, Microbiology, Biochemistry and Immunology, and Clinical Research Center, Morehouse School of Medicine, Atlanta, GA 30310, USA; 3 Departments of Epidemiology and Health Services Research, University of Florida, Gainesville, FL 32611, USA; 4 Department of Organization and Management, University of California, Irvine, CA 92697, USA; 5 National Center for Advancing Translational Sciences, National Institutes of Health, Rockville, MD 20850, USA; 6 Council of Graduate Schools, Washington, DC 20036, USA

**Keywords:** COVID-19, early-stage investigators, developmental networks, grant writing coaching, mentoring

## Abstract

The COVID-19 pandemic has significantly taxed scientific research and seems to have exacerbated existing inequities within the research field, particularly for early-stage investigators (ESIs). This study examines the effects of the COVID-19 pandemic on traditionally underrepresented ESIs enrolled in an NIH-supported study evaluating the effectiveness of developmental networks, grant writing coaching, and mentoring on research career advancement. The survey consisted of 24 closed-ended (quantitative) and 4 open-ended questions (qualitative) linked to a participant’s ability to meet grant submission deadlines, research and professional development disruptions, stress level, career transition level, self-efficacy and management of scholarly tasks, and familial responsibilities. Results from 32 respondents (53%) suggest that COVID-19 adversely impacted the continuity of research (81%) and grant submissions (63%). On average, grant submissions were delayed by 6.69 months (i.e., greater than one grant cycle). We also conducted additional analyses characterizing nonresponse and found that there were no significant predictors of nonresponse, indicating a limited threat to the validity of our findings. The disruption caused by COVID-19 to the careers of ESIs from underrepresented groups in the biomedical workforce has been profound in the short term. The long-term consequences to the future success of these groups are unknown but is a worthwhile area of research and potential innovation.

## 1. Introduction

COVID-19 negatively impacted human health, economics, education, and research worldwide [1,2,3,4,5,6,7]. Strategies to mitigate the spread of COVID-19 paused most laboratory and clinical research. Research training and faculty position openings for early-stage investigators (ESIs) were interrupted [6,8]. Lockdowns to fight the pandemic forced universities to switch rapidly to distance learning. Faculty, driven into unplanned online teaching, had to invest extra time into developing their online teaching syllabi. This shift has been particularly challenging for women and racial/ethnic minorities, since they are usually more likely to experience heavy teaching loads [8,9,10,11,12,13,14,15,16,17].

Early-stage investigators (ESIs), especially underrepresented minorities and female researchers, are at the highest risk of experiencing a negative impact of COVID-19 on their career advancement [9,13,15,16,17]. For example, the immediate impact of the COVID-19 pandemic was high on ESIs participating in two other NIH-sponsored career development programs, KL2 and TL1 [18]. The KL2 program trains clinically focused ESIs to succeed in patient-oriented clinical research. Results of their survey show that the most negative impacts on KL2 scholars were 1. lack of access to human subjects (69%), core facilities (55%), and laboratories (51%), and 2. loss of work time because of having to homeschool their children (44%). The TL1 program provides training and career development for pre- and postdoctoral scholars to become translational science researchers. Results of those surveyed show that 25% of TL1 trainees stopped their training and career development activities. They reported limited access to laboratories, seminars, and workshops, a lack of clinical exposure, and less engagement with mentors.

ESIs’ commitment to biomedical research careers has been positively associated with mentorship [19,20,21]. The success of new investigators is dependent on securing career-facilitating mentoring [22,23]. Mentorship is essential for ESIs from diverse groups, including women and minorities, as these groups continue to be underrepresented in biomedical and behavioral research in the United States [22,23,24]. Ginther et al. [25] confirmed the disparity between racial minority groups and their White counterparts regarding receiving NIH research funding. One conclusion of their results is that mentoring and coaching are significant determinants of success in gaining grant awards. 

Our group sought to address this grant and career development “gap” by employing a National Research Mentoring Network (NRMN) project, “Randomized Controlled Study to Test the Effectiveness of Development Network (DN) Coaching in the Career Advancement of Diverse Early-Stage Investigators” (NIGMS 5U01GM132771) [26,27]. The DN intervention provides a framework for measuring and testing the contribution of network connections to progress in reaching desired levels of research productivity and career advancement. The DN intervention centers on the scholar’s social capital and support by developers, peers, and mentors [28,29,30]. 

Our UO1 study evaluates ESIs’ progress toward research independence by focusing on grant writing, networking skills, and the technical aspects of pursuing research independence. This paper focuses on the extent to which the COVID-19 pandemic impacted the scholarly progress of the first cadre of diverse ESIs participating in our study. We hypothesized that the ESIs from underrepresented groups enrolled in our study might be most vulnerable to disruptions from the COVID-19 pandemic. 

## 2. Materials and Methods

### 2.1. Survey Design

We conducted an online survey to gain insight into disruptions experienced during the COVID-19 pandemic by the first cohort of scholars in the parent U01 study. The U01 study is framed as the Strategic Empowerment Tailored for Health Equity Investigators (NRMN-SETH) based on Grant Writing and Coaching Groups implemented in NRMN Phase I [27,31,32]. The participants that went through a competitive application process were ESIs, some were not from underrepresented groups but were working in minority institutions, focusing on health equity research and randomly assigned to receive structured grant writing coaching (Group A) or mentoring around developmental networks and structured grant writing coaching (DN intervention/Group B) [33]. The DN intervention is delivered remotely via a technology platform that supports synchronous and asynchronous peer and coach interactions [28,29,30]. Cohort 1, this report’s focus, started in December 2019. We collected basic demographic and professional details (i.e., participants’ gender, ethnicity, field of study, career stage, and faculty rank) via REDCap. The Morehouse School of Medicine IRB approved this research under an IRB Authorization Agreement (IRB Approval #1352302-3). 

The survey, which sought to understand the scholars’ assessments of COVID-19’s impact on their career trajectories, was administered between July and August 2020. The survey included 24 multiple-choice, Likert-scale, and 4 open-ended questions that allowed the gathering of quantitative and qualitative data. The survey questions were developed by the NRMN COVID Working Group [34,35] and shared with all NRMN UO1 awardees. These included questions about the impact of COVID-19 on career trajectory, training trajectory, mentoring, life impact, and social unrest. Questions were selected after discussion among the study researchers. Open-ended questions asked scholars to reflect on their experiences during COVID-19. Our goal was to identify specific areas of impact, including the participant’s ability to meet grant submission deadlines and sustain communication with coaches/developers, to manage stress levels, career transition level, self-efficacy, and scholarly tasks, and build overall confidence to meet career challenges, and the effects of their family situation on their scholarly progress. 

Scholars represented various minority-serving institutions with Research Centers in Minority Institutions (RCMI), Institutional Development Award (IDeA) programs, and research institutions with Clinical and Translational Science Award (CTSA) programs. The survey was not intended to collect data representative of all ESIs, and, therefore, should not be extrapolated as quantitative estimates to all ESIs.

### 2.2. Data Analysis

Descriptive statistics were used to describe the respondents’ characteristics and responses in terms of frequencies and percentages. To ensure that nonresponse bias did not threaten the validity of the findings, we used a logistic regression to analyze whether a participant responded to the survey as a function of respondent-specific characteristics, (i.e., gender, whether the respondent dropped out of the study, institution type, randomization group, career stage, research category, perception of institutional support, and teaching load).

Manual coding of open-ended questions was performed because it was a small sample (4 questions and a maximum of 32 responders) and nothing was likely to be lost. One author conducted the coding, independently analyzing the responses. Answers were read and assigned to categories based on the content and specific words. Through a deductive process, themes related to the impact of the pandemic on mentorship, research career, training trajectory, and academic life were identified. A second author reviewed the coding and agreed with the codes since the answers were brief and open. Inter-rater reliability (IRR) was not calculated because one author conducted the coding on a small specific sample, and other authors reviewed and discussed the codes and themes [36,37]. 

## 3. Results

### 3.1. Scholars’ Characteristics

A total of 60 participants recruited to Cohort 1 were randomized to Group A (29 participants) or Group B (31 participants). Table 1 summarizes the scholar’s characteristics. Altogether, 42 (70%) were female, and the majority self-identified as an ethnically or racially underrepresented minority (URM): 25 (42%) African American, 13 (22%) Latinx, 10 (15%) Asian, 8 (13%) White, and 4 (7%) other. As for their career stage, 44 were assistant professors (73%), 2 were associate professors (3%), 3 were instructors (5%), 10 were postdoctoral trainees (17%), and 1 was other (a scientist in a non-academic setting) (1.7%). Their current teaching load varied from teaching three courses per academic term (7%) to no teaching (15%). Most (27%) taught two courses per academic term. Scholars represented research fields across the biomedical spectrum, with 38% from social or behavioral sciences research, 37% in clinical and/or translational research, and 25% in basic sciences/biomedical research. Regarding the proposals in preparation, 32 (53%) scholars were developing R-series grant proposals, 23 (38%) were developing K-award proposals, and 5 were applying for other types of awards. Thirteen participants dropped out before March 2020, when academic institutions moved classes online (five from Group A, eight from Group B). Nine participants dropped out after March 2020 (four from Group A, five from Group B). However, the survey was sent to all participants enrolled in the U01 study, and 32 responses in total were collected (a response rate of 53%). Statistically insignificant results indicate that there were no significant predictors of nonresponse, as shown in Table 1.

### 3.2. Survey Results

Table 2 presents the survey results on the impact of the COVID-19 pandemic on the Cohort 1 of NRMN-SETH. Altogether, 28 of 32 (90%) respondents indicated that the COVID-19 crisis impacted their ability to perform research, and, overall, 24 (75%) had interrupted experiments critical to their proposals. COVID-19 affected the anticipated grant application submission date for 20 (63%) of the respondents. Twenty-four (75%) continued to communicate with their grant writing coaches online. Interestingly, 15 (47%) were able to incorporate COVID-19 studies into their research portfolios. These new research opportunities included a nanoparticle delivery method for a SARS-CoV-2 vaccine, evaluating oral health in COVID-19 patients and evaluating resilience factors associated with COVID-19. A majority of scholars felt unable to overcome difficulties arising due to the pandemic. A total of 24 (75%) reported difficulty in maintaining concentration on their work; 12 (38%) reported increased pandemic-related financial stress; 4 (13%) reported increased racial discrimination; and 13 (41%) were separated from their families. 

Overall, 24 of 32 (75%) respondents indicated that their workload had increased. A total of 21 (66%) respondents reported work disruption due to increased familial responsibilities (e.g., childcare), including 15 of 18 females and 6 of 14 males. Finally, seven (22%) had an increase in writing productivity while in lockdown.

### 3.3. Themes in Open-Ended Questions

#### 3.3.1. Academic Life/Teaching

Individual accounts of the pandemic’s impact show clear signs of stress and reaffirm the answers to closed-ended questions. The COVID-19 pandemic caused a dramatic increase in teaching time. Scholars devoted most of their time to developing online courses. Many had never designed or delivered a course online; thus, they had to adjust to fully online teaching in a short time and with limited guidance.

“*With the format changed for classes, I had to change all my classes. In addition, I have to learn how to use different platforms like blackboard, CANVAS, Zoom, Teams, etc. for my classes and meetings. My supervisors organized meetings almost daily. With all these I do not have time to seek for mentorship. I need time and my own space to continue with my proposal*.”

“*Has slowed things down due to major focus on preparing for teaching and learning new tools, etc. for effectively teaching online*.”

The pressures put on the faculty by COVID-19 were further exacerbated due to hiring freezes instituted by universities and colleges. Many interviews for open positions were canceled and pushed the teaching responsibilities back on the current faculty.

“*Interview process was canceled*.”

“*Unfortunately, I’ve had to decrease my research time and increase my time converting my in-class teaching to online*.”

#### 3.3.2. Research Productivity/Career Development/Mentoring

Scholars could not meet the timeframe to complete their proposals and mentorship plans and had to extend the submission deadline date. The responses show several contributing factors to these delays, including technical, human, and research resources.

“*without access to campus and my resources on campus, such as software/printer and colleagues, it is difficult to maintain productivity and efficiency.”*

“*I delayed my planned R01 submission for 6 months on my current research. Many of my research projects in the lab have been delayed by 3 months due to limited access to the research lab. I’ve also had to delay multiple manuscripts because I’ve had insufficient amounts of time to complete the research and the writing*.”

Another disruption came from the need to re-align meeting schedules and communication channels with peers and mentors.

“*The pandemic has interrupted my communication with others and the timeline for accomplishing my career goals*.”

“*It’s greatly impacted my mentoring schedule and productivity to meet goals in the training trajectory*.”

“*I haven’t sought out mentorship, just trying to keep afloat.*”

Collection of preliminary data, ongoing experiments, and access to research populations also stopped. Scholars involved in studies including human subjects were negatively affected by COVID-19 since in-person interactions were paused. This effect was reported for both downstream, ongoing, and previously funded studies and the upstream, pilot, and preliminary data collection.

“*ongoing experiments had to be discarded, we were collecting preliminary data for grant application*.”

“*I had intended to collect pilot data for a grant submission but am unable to given that my population interest is older adults*.”

“*There is community-engaged research I am finishing up that I haven’t been able to finish because of the pandemic*.”

Some scholars were successful in shifting their research to aspects of COVID-19. NIH and other agencies issued calls for supplements to existing grants, and several scholars were able to pivot their efforts to capitalize on these opportunities. Others were able to identify new research questions that came to importance because of the pandemic.

“*I was funded through a supplement to investigate COVID-19 disparities among communities of color in the US*.”

“*I wrote a commentary that is conditionally accepted by Public Health Reports and am joining a qualitative Covid project at my new institution*.”

“*I am now working on resilience factors related to COVID-19*.”

#### 3.3.3. Family Life /Caregiving

The impact of COVID-19 was most pronounced for women scientists with young dependents.

“*Since I have 2 children at home, I’ve had to reevaluate my priorities with regards to work and life*.”

“*I just need the world to stop so I can catch up. It’s hard to think about doing “regular work” when children are stuck at home and parents are stuck in nursing homes*.”

“*I’m the primary caretaker for my 2 children (ages 6 and 8), which makes it hard to complete my work due to constant interruptions at home*.”

“*When COVID happened and I lost childcare, I just felt like I couldn’t continue with the program. I feel so guilty…”*

#### 3.3.4. Grief/Stress

Lastly, the COVID-19 has been very taxing emotionally for all scholars. Isolation measures brought to the forefront the importance of connection, interpersonal relations, and social capital. Scholars felt that access to many resources that were available to them had vanished. They saw the NRMN-SETH as a critical career development resource but also realized that access to this program and network is limited by the timeline of each cohort. The notions of a timeline and the need to stop time were also common. Finally, most of the NRMN-SETH participants came from racial and ethnic minority groups, and all participants demonstrated a strong commitment to promoting diversity through health equity research. The national events and the Black Lives Matter movement were a common reference among their comments, and several directly linked these events to the added stress, anxiety, and lack of connection they experienced.

“*I am most in need of regular check ins with faculty and students. The moments of passing in the hallway, unscheduled connection. I am also in need of counseling to help manage the constant stress of the pandemic and handling my responsibilities. I am also feeling more burned out than usual*.”

“*I am hoping that the SETH NRMN program deadline will be extended. We can and will get through this!*”

“*Combination of the pandemic and black lives matter protests have made life very stressful. I just don’t know what to expect anymore*.”

“*There were no resources to me. This has been a very difficult time and one of the lowest points in my life*.”

## 4. Discussion

The devastating effect of the COVID-19 epidemic on education and research training worldwide is extensive and its impact has lasted, as recent publications show [5,6,18,38]. Our study offers an important perspective for the field because our cohort of ESIs provide a unique perspective. Most are young, full-time teaching faculty, predominantly minority scientists who are historically underrepresented in biomedical research, from under-resourced institutions and who are highly motivated to succeed in research addressing health equity research. Our study found that COVID-19 impacted respondents’ ability to meet grant submission deadlines and perform research. They faced unprecedented challenges, had to prioritize familial responsibilities, and concurrently learned to use new technologies to meet new demands as online educators. There was a significant decline in the time they devoted to research and grant writing; consequently, their research career development was substantially affected. One significant consequence is that the submission of grant proposals being developed in our study was delayed by one grant cycle. Respondents perceived that their ability to devote time to their research careers was substantially affected. In addition, qualitative comments indicate that transitioning to teaching online caused delays in grant submission timelines. The significant impact of familial responsibilities on the research career of female investigators was consistent with research by others that highlights a persistent gender gap in science [7,15,16,38], which is most pronounced for women scientists with young dependents [5,7,9,38]. In general, women scholars carry the load of homeschooling responsibilities, which decreases the time they have available to dedicate to scholarly productivity. Traditionally, childcare falls on female members of the household, so while male ESIs in our study perceived that they had to take on more responsibility for these tasks because of the pandemic, the female ESIs still faced most of this burden [7,38]. 

COVID-19 has uprooted the existing integration and social capital accessible to ESIs, has broken the traditional means of engagement within scholarly communities, and challenged faculty development practices. Restrictions imposed by COVID-19 made communication between grant leaders, mentors, research advisors, and ESIs more challenging. In our study, a high percentage (76%) of the respondents were able to keep in touch with coaches and developers, especially considering competing priorities due to COVID-19 pandemic. The NRMN-SETH technology platform that supports synchronous and asynchronous peer and coach interactions helped shape their personal and career progress and was described as having been of great value and to have provided them social support. Whereas faculty meetings and face-to-face workshops had previously promoted network development and professional collaboration, under the COVID-19 isolation protocols, these serendipitous connections were no longer possible. It took time to adopt new faculty development practices that are both acceptable and effective for the academic community. The NRMN-SETH experience is an example of one such practice. It focuses on grant writing coaching and the development of professional networks. The NRMN-SETH cohort and small group model create active peer connections and provide regular, structured access to social capital. Peers and coaches collaboratively act as a resource for each other whilst remaining geographically dispersed, and new types of connections are formed. This experience supports two propositions: first, that structured interventions at cohort and small group levels are effective in achieving coherence, a sense of belonging, and fostering academic community development, and second, that trans-disciplinarity supports a cohort model of faculty development through exposure to diverse backgrounds and cultural perspectives.

### Limitations

Despite our best attempt to show no systematic differences between participants who completed the survey compared to nonresponders, our sample may not be representative of all the ESIs participating in our study. For example, the ESIs most affected by the pandemic may have been the ones most likely to respond. The limited sample size and consequential statistical power precluded meaningful analyses of the effect of modifying covariates such as gender and increased familial responsibilities on the impact of COVID-19 on research productivity and grant writing. Other limitations may also pertain to the scope of the survey as it was focused on a training program and may not be generalizable to ESIs in other contexts. The participants chose to respond to the survey and were, therefore, self-selected, which may also limit the generalizability of our findings.

## 5. Conclusions

Disruptions caused by COVID-19 to careers of underrepresented ESIs in the biomedical workforce have been profound in the short term, as judged by this study. Future studies and longer-term follow-ups are needed to determine the impact of such drastic and sustained interruptions on the retention of these scholars from underrepresented groups in biomedical research. The role of a virtual approach in building resiliency in ESIs’ future research should be evaluated further. We found areas for institutional consideration that could create or enhance a climate that supports the success of underrepresented ESIs scholarship. One is to support faculty in developing schedules that work alongside the institution’s and students’ needs while prioritizing faculty scholarship. Another is to establish or increase the number of programs that promote the faculty’s well-being and work-life integration. Studies should also examine novel potential policy measures and interventions by the National Institutes of Health and other funding agencies to support underrepresented ESI scholarship.

## Figures and Tables

**Table 1:** Characteristics of Cohort 1 Scholars and Survey Response Rates.

	All		Respondents		Non-Respondents		
	N	(%)	N	(%)	N	(%)	*p* Value
***Gender***							
Female	42	70.00%	23	71.88%	19	67.86%	-
Male	18	30.00%	9	28.13%	9	32.14%	0.12
***Race/Ethnicity***							
African American (Black)	25	41.67%	7	21.88%	18	64.29%	-
Asian	10	16.67%	5	15.63%	5	17.86%	-
Hispanic or Latino/Latina	13	21.67%	9	28.13%	4	14.29%	-
White	8	13.33%	8	25.00%	0	0.00%	-
Other	4	6.67%	3	9.38%	1	3.57%	-
***Career Stage***							
Associate professor	2	3.33%	1	3.13%	1	3.57%	0.33
Assistant professor	44	73.33%	26	81.25%	18	64.29%	-
Instructor	3	5.00%	1	3.13%	2	7.14%	0.34
Postdoctoral associate/fellow	10	16.67%	3	9.38%	7	25.00%	0.13
Other (scientist in non-academic setting)	1	1.67%	1	3.13%	0	0.00%	0.99
***Research Category***							
Biomedical	15	25.00%	8	25.00%	7	25.00%	-
Clinical and/or translational	22	36.67%	13	40.63%	9	32.14%	0.97
Social/behavioral science	23	38.33%	11	34.38%	12	32.86%	0.68
***Funding Mechanism Sought in NRMN Grant Writing Program***							
R-type mechanism	32	53.33%	20	62.50%	12	42.86%	-
K-type mechanism	23	38.33%	9	28.13%	14	50.00%	-
NSF research grant	1	1.67%	1	3.13%	0	0.00%	-
Other	4	6.67%	2	6.25%	2	7.14%	-
***What is your current teaching load?***							
Occasional teaching and lecture	19	31.67%	10	31.25%	9	32.14%	-
One course per academic term	10	16.67%	6	18.75%	4	14.29%	-
Two courses per academic term	16	26.67%	10	31.25%	6	21.43%	-
Three courses per academic term	4	6.67%	1	3.13%	3	10.71%	-
More than three courses	2	3.33%	2	6.25%	0	0.00%	-
Not applicable (not teaching)	9	15.00%	3	9.38%	6	21.43%	-

**Table 2:** The Impact of COVID-19 Pandemic on Cohort 1 of NRMN-SETH.

	All	Male	Female
**COVID-19 crisis impacted my ability to perform research**			
Yes	28 (90.00%)	7 (%)	21 (%)
No	3 (9.38%)	2 (%)	1 (%)
Missing	1 (3.13%)	-	-
**Experiments critical to my research and proposal were interrupted**			
Yes	24 (75.00%)	5 (%)	19 (%)
No	8 (25%)	4 (%)	4 (%)
**COVID-19 affected my anticipated grant application submission date**			
Yes	20 (62.50%)	5 (%)	15 (%)
No	12 (37.50%)	4 (%)	8 (%)
**During the pandemic I continued to communicate with my grant writing coaches online**			
Yes	24 (75.00%)	8	16
No	8 (25.00%)	1	7
**I was able to incorporate COVID-19 into my research portfolio**			
Yes	15 (46.88%)	4 (%)	11 (%)
No	17 (53.13%)	5 (%)	12 (%)
**I felt unable to overcome difficulties due to the pandemic**			
Never	1 (3.33%)	0 (0.00%)	1 (100.00%)
Almost Never	6 (18.75%)	2 (33.00%)	4 (66.67%)
Sometimes	8 (25.00%)	2 (25.00%)	6 (75.00%)
Fairly Often	14 (43.75%)	5 (35.71%)	9 (64.29%)
Very Often	3 (9.38%)	0 (0.00%)	3 (100.00%)
**During the pandemic I experienced difficulties concentrating**			
Yes	24 (75.00%)	6 (25.00%)	18 (75.00%)
No	8 (25.00%)	3 (37.50%)	5 (62.50%)
**During the pandemic I experienced increased pandemic-related financial stress**			
Yes	12 (37.50%)	3 (25.00%)	9 (75.00%)
No	20 (62.50%)	6 (30.00%)	14 (70.00%)
**During the pandemic I experienced increased racial discrimination**			
Yes	4 (12.50%)	1 (25.00%)	3 (75.00%)
No	28 (87.50%)	8 (28.57%)	20 (71.43%)
**During the pandemic I was separated from family and friends (Quarantining alone)**			
Yes	13 (40.63%)	1 (7.69%)	12 (92.31%)
No	19 (59.38%)	8 (42.11%)	11 (57.89%)
**During the pandemic my workload had increased**			
Yes	24 (75.00%)	7 (29.17%)	17 (70.83%)
No	8 (25.00%)	2 (25.00%)	6 (75.00%)
**During the pandemic, I experienced family related disruptions to work (e.g., childcare needs in home, helping child with schoolwork, caring for family members)**			
Yes	21 (65.63%)	6 (28.57%)	15 (71.43%)
No	11 (34.38%)	8 (72.73%)	3 (27.27%)
**During the pandemic I experienced an increase in writing productivity while in lockdown**			
Yes	7 (21.88%)	2 (28.57%)	5 (71.43%)
No	25 (78.13%)	7 (28.00%)	18 (72.00%)

## Data Availability

The data presented in this study are available on request from the corresponding author. The data are not publicly available due to privacy concerns.

## References

[1] Pak A., Adegboye O.A., Adekunle A.I., Rahman K.M., McBryde E.S., Eisen D.P. (2020). Economic Consequences of the COVID-19 Outbreak: The Need for Epidemic Preparedness. Front. Public Health.

[2] Veletsianos G., Houlden S. Coronavirus Pushes Universities to Switch to Online Classes—But Are They Ready?. http://theconversation.com/coronavirus-pushes-universities-to-switch-to-online-classes-but-are-they-ready-132728.

[3] Myers K.R., Tham W.Y., Yin Y., Cohodes N., Thursby J.G., Thursby M.C., Schiffer P., Walsh J.T., Lakhani K.R., Wang D. (2020). Unequal Effects of the COVID-19 Pandemic on Scientists. Nat. Hum. Behav..

[4] Coyne C., Ballard J.D., Blader I.J. (2020). Recommendations for Future University Pandemic Responses: What the First COVID-19 Shutdown Taught Us. PLoS Biol..

[5] Cardel M.I., Dean N., Montoya-Williams D. (2020). Preventing a Secondary Epidemic of Lost Early Career Scientists. Effects of COVID-19 Pandemic on Women with Children. Ann. Am. Thorac. Soc..

[6] Kent D.G., Knapp D.J.H.F., Kannan N. (2020). Survey Says: “COVID-19 Lockdown Hits Young Faculty and Clinical Trials”. Stem Cell Rep..

[7] Higginbotham E., Dahlberg M.L. (2021). The Impact of COVID-19 on the Careers of Women in Academic Sciences, Engineering, and Medicine.

[8] Universities Are Freezing Tenure Clocks What Will That Mean for Junior Faculty of Color?. https://www.higheredjobs.com/Articles/articleDisplay.cfm?ID=2238.

[9] Minello A. (2020). The Pandemic and the Female Academic. Nature.

[10] Jacobs L., Cintrón J., Canton C.E. (2002). The Politics of Survival in Academia: Narratives of Inequity, Resilience, and Success.

[11] Porter S.R. (2007). A Closer Look at Faculty Service: What Affects Participation on Committees?. J. High. Educ..

[12] Joseph T.D., Hirshfield L.E. (2011). ‘Why Don’t You Get Somebody New to Do It?’ Race and Cultural Taxation in the Academy. Ethn. Racial Stud..

[13] Rodríguez J.E., Campbell K.M., Pololi L.H. (2015). Addressing Disparities in Academic Medicine: What of the Minority Tax?. BMC Med. Educ..

[14] Kaplan S.E., Raj A., Carr P.L., Terrin N., Breeze J.L., Freund K.M. (2018). Race/Ethnicity and Success in Academic Medicine: Findings From a Longitudinal Multi-Institutional Study. Acad. Med. J. Assoc. Am. Med. Coll..

[15] Carson T.L., Aguilera A., Brown S.D., Peña J., Butler A., Dulin A., Jonassaint C.R., Riley I., Vanderbom K., Molina K.M. (2019). A Seat at the Table: Strategic Engagement in Service Activities for Early-Career Faculty From Underrepresented Groups in the Academy. Acad. Med. J. Assoc. Am. Med. Coll..

[16] Krukowski R.A., Jagsi R., Cardel M.I. (2021). Academic Productivity Differences by Gender and Child Age in Science, Technology, Engineering, Mathematics, and Medicine Faculty During the COVID-19 Pandemic. J. Womens Health 2002.

[17] Malisch J.L., Harris B.N., Sherrer S.M., Lewis K.A., Shepherd S.L., McCarthy P.C., Spott J.L., Karam E.P., Moustaid-Moussa N., Calarco J.M. (2020). Opinion: In the Wake of COVID-19, Academia Needs New Solutions to Ensure Gender Equity. Proc. Natl. Acad. Sci. USA.

[18] McCormack W.T., Bredella M.A., Ingbar D.H., Jackson R.D., Meagher E.A., Morris C.D., Nagel J.D., Pusek S., Rubio D.M., Sandberg K. (2020). Immediate Impact of the COVID-19 Pandemic on CTSA TL1 and KL2 Training and Career Development. J. Clin. Transl. Sci..

[19] The Role of Efficacy and Identity in Science Career Commitment Among Underrepresented Minority Students—Chemers—2011—Journal of Social Issues—Wiley Online Library. https://spssi.onlinelibrary.wiley.com/doi/abs/10.1111/j.1540-4560.2011.01710.x.

[20] McGinn A.P., Lee L.S., Baez A., Zwanziger J., Anderson K.E., Seely E.W., Schoenbaum E. (2015). Mentoring in Clinical-Translational Research: A Study of Participants in Master’s Degree Programs. Clin. Transl. Sci..

[21] McGee R. (2016). Biomedical Workforce Diversity: The Context for Mentoring to Develop Talents and Foster Success within the “Pipeline.”. AIDS Behav..

[22] Lambert W.M., Wells M.T., Cipriano M.F., Sneva J.N., Morris J.A., Golightly L.M. (2020). Career Choices of Underrepresented and Female Postdocs in the Biomedical Sciences. eLife.

[23] Dahlberg M.L., Byars-Winston A., National Academies of Sciences, Engineering, and Medicine, Policy and Global Affairs, Board on Higher Education and Workforce, Committee on Effective Mentoring in STEMM (2019). The Science of Effective Mentorship in STEMM.

[24] Ginther D.K., Schaffer W.T., Schnell J., Masimore B., Liu F., Haak L.L., Kington R.S. (2009). Diversity in Academic Biomedicine: An Evaluation of Education and Career Outcomes with Implications for Policy (22 September 2009). https://ssrn.com/abstract=1677993.

[25] Ginther D.K., Schaffer W.T., Schnell J., Masimore B., Liu F., Haak L.L., Kington R. (2011). Race, Ethnicity, and NIH Research Awards. Science.

[26] National Institute of General Medical Sciences. https://nigms.nih.gov/.

[27] Sorkness C.A., Pfund C., Ofili E.O., Okuyemi K.S., Vishwanatha J.K., Zavala M.E., Pesavento T., Fernandez M., Tissera A., NRMN Team (2017). A New Approach to Mentoring for Research Careers: The National Research Mentoring Network. BMC Proc..

[28] Higgins M.C., Thomas D.A. (2001). Constellations and Careers: Toward Understanding the Effects of Multiple Developmental Relationships. J. Organ. Behav..

[29] Yip J., Kram K.E. (2017). The SAGE Handbook of Mentoring. The SAGE Handbook of Mentoring.

[30] Hall M., Engler J., Hemming J., Alema-Mensah E., Baez A., Lawson K., Quarshie A., Stiles J., Pemu P., Thompson W. (2018). Using a Virtual Community (the Health Equity Learning Collaboratory) to Support Early-Stage Investigators Pursuing Grant Funding. Int. J. Environ. Res. Public. Health.

[31] Hemming J.J., Baez A., Hall M., Thompson W., Stiles J., Ofili E. (2019). Advancing Health Equity through Organizational Mentoring Policies at Minority- Serving Institutions. Ethn. Dis..

[32] Weber-Main A.M., McGee R., Eide Boman K., Hemming J., Hall M., Unold T., Harwood E.M., Risner L.E., Smith A., Lawson K. (2020). Grant application outcomes for biomedical researchers who participated in the National Research Mentoring Network’s Grant Writing Coaching Programs. PLoS ONE.

[33] Mubasher M., Lawson K., Pemu P., Pearson T., Engler J., Baez A., Stiles J.K., Salazar M.S., Caplan L.S., Green K. (2021). Randomized Controlled Study to Test the Effectiveness of Developmental Network Coaching in the Career Advancement of Diverse Early-Stage Investigators (ESIs): Implementation Challenges and Lessons Learned. Int. J. Environ. Res. Public. Health.

[34] Cohen S., Kamarck T., Mermelstein R. (1983). A global measure of perceived stress. J. Health Soc. Behav..

[35] Byars-Winston A., Leverett P., Benbow R.J., Pfund C., Thayer-Hart N., Branchaw J. (2020). Race and Ethnicity in Biology Research Mentoring Relationships. J. Divers. High. Educ..

[36] Sutton J., Austin Z. (2015). Qualitative Research: Data Collection, Analysis, and Management. Can. J. Hosp. Pharm..

[37] McDonald N., Schoenebeck S., Forte A. (2019). Reliability and Inter-rater Reliability in Qualitative Research: Norms and Guidelines for CSCW and HCI Practice. Proc. ACM Hum.-Comput. Interact..

[38] Tugend A. On the Verge of Burnout: COVID-19′s Impact on Faculty Wellbeing and Career Plans Generated by the Chronicle of Higher Ed in Collaboration with Fidelity Investments. https://connect.chronicle.com/rs/931-EKA218/images/Covid%26FacultyCareerPaths_Fidelity_ResearchBrief_v3%20%281%29.pdf.

